# Incidence of lung cancer histologic cell-types according to neighborhood factors: A population based study in California

**DOI:** 10.1371/journal.pone.0197146

**Published:** 2018-05-23

**Authors:** Mindy C. DeRouen, Lauren Hu, Meg McKinley, Kathleen Gali, Manali Patel, Christina Clarke, Heather Wakelee, Robert Haile, Scarlett Lin Gomez, Iona Cheng

**Affiliations:** 1 Cancer Prevention Institute of California, Fremont, CA, United States of America; 2 Department of Epidemiology and Biostatistics, University of California San Francisco, San Francisco, CA, United States of America; 3 Greater Bay Area Cancer Registry, Fremont, CA, United States of America; 4 John A. Burns School of Medicine, University of Hawaii, Honolulu, HI, United States of America; 5 Department of Public Health, School of Social Sciences, Humanities and Arts, University of California Merced, Merced, CA, United States of America; 6 Division of Oncology, Department of Medicine, Stanford University School of Medicine, Stanford, CA, United States of America; 7 Veterans Affairs Palo Alto Health Care System, Palo Alto, CA, United States of America; 8 Primary Care and Outcomes Research, Department of Medicine, Stanford University School of Medicine, Stanford, CA, United States of America; 9 Stanford Cancer Institute, Stanford, CA, United States of America; 10 Department of Medicine, Cedars-Sinai Medical Center, Los Angeles, CA, United States of America; 11 UCSF Helen Diller Family Comprehensive Cancer Center, San Francisco, CA, United States of America; Leibniz Institute for Prevention Research and Epidemiology BIPS, GERMANY

## Abstract

**Background:**

The relationships between neighborhood factors (i.e., neighborhood socioeconomic status (nSES) and ethnic enclave) and histologic subtypes of lung cancer for racial/ethnic groups, particularly Hispanics and Asian American/Pacific Islanders (AAPIs), are poorly understood.

**Methods:**

We conducted a population-based study of 75,631 Californians diagnosed with lung cancer from 2008 through2012. We report incidence rate ratios (IRRs) for lung cancer histologic cell-types by nSES among racial/ethnic groups (non-Hispanic (NH) Whites, NH Blacks, Hispanics and AAPIs) and according to Hispanic or Asian neighborhood ethnic enclave status among Hispanics and AAPIs, respectively. In addition, we examined incidence jointly by nSES and ethnic enclave.

**Results:**

Patterns of lung cancer incidence by nSES and ethnic enclave differed across race/ethnicity, sex, and histologic cell-type. For adenocarcinoma, Hispanic males and females, residing in both low nSES and high nSES neighborhoods that were low enclave, had higher incidence rates compared to those residing in low nSES, high enclave neighborhoods; males (IRR, 1.17 [95% CI, 1.04–1.32] and IRR, 1.15 [95% CI, 1.02–1.29], respectively) and females (IRR, 1.29 [95% CI, 1.15–1.44] and IRR, 1.51 [95% CI, 1.36–1.67], respectively). However, AAPI males residing in both low and high SES neighborhoods that were also low enclave had lower adenocarcinoma incidence.

**Conclusions:**

Neighborhood factors differentially influence the incidence of lung cancer histologic cell-types with heterogeneity in these associations by race/ethnicity and sex. For Hispanic males and females and AAPI males, neighborhood ethnic enclave status is strongly associated with lung adenocarcinoma incidence.

## Introduction

Lung cancer is the second-most common cancer among both males and females in the U.S. with over 222,500 new cases of lung cancer estimated to be diagnosed in 2017 [1]. Lower area-level socioeconomic status (SES) has been associated with a higher incidence of overall lung cancer and non-small-cell lung cancer (NSCLC) among non-Hispanic White and non-Hispanic Black males and females and Asian American/Pacific Islander (AAPI) males [2–5]. Disparities in the burden of lung cancer according to area-level SES may be mediated by higher smoking prevalence, lower access to and use of health care services, and/or greater exposure to environmental contaminants such as air pollution, radon, or asbestos among those residing in lower SES neighborhoods [6–10]. On the other hand, lower area-level SES has been associated with lower incidence of overall lung cancer and NSCLC among Hispanic males and females [2, 4, 5]. The reason for this paradoxical association among Hispanics is unknown, but the ethnic composition of neighborhoods, rather than SES, has been suggested to play a key role [11]. Finally, while incidence of overall lung cancer and NSCLC according to area-level SES has been examined among AAPI females, no patterns have been reported [2, 4, 5] and other neighborhood factors such as Asian enclave have not yet been examined among AAPIs.

Lung cancer is divided into two broad histologic cell type categories: small cell lung carcinoma (SCLC) and NSCLC, the latter of which is further divided into adenocarcinoma, squamous cell carcinoma (SCC), and large cell or other specified carcinoma (LC+OSC) subtypes. While prior studies have described incidence of overall lung cancer or NSCLC according to neighborhood factors, none have reported incidence according to types of NSCLC. Histologic cell types, including types of NSCLC, differ in their association with cigarette smoking and nonsmoking-related risk factors [12, 13]. For example, cigarette smoking is the strongest risk factor for every histologic cell-type of lung cancer, but the strength of this association differs by histologic cell-type; it has been reported that SCC and SCLC are more strongly associated with smoking compared to adenocarcinoma and LC+OSC [12]. It stands to reason that disentangling the variation in the incidence of histologic cell types according to neighborhood factors may provide insight into possible etiologic differences across racial/ethnic groups and may highlight vulnerable subpopulations at high risk. Therefore, we conducted a large, contemporary population-based study including 75,631 Californians diagnosed from 2008 through 2012 to compare incidence of lung cancer histologic cell-types according to neighborhood SES (nSES) and ethnic enclave status.

## Materials and methods

### Study population

The study population included all incident first primary cases of lung cancer (n = 75,631) diagnosed from January 2008 through December 2012. Cases were identified by the California Cancer Registry (CCR), part of the National Cancer Institute’s Surveillance, Epidemiology, and End Results (SEER) program. The study was conducted with IRB oversight from the Cancer Prevention Institute of California. Data on age at cancer diagnosis, sex, race/ethnicity, residential address at diagnosis, and tumor histologic cell type and stage (SEER Summary Stage) were obtained. Histologic cell-types were defined according to Lewis et al. [14] for small-cell lung cancer (SCLC), adenocarcinoma, squamous cell carcinoma (SCC), large cell and other specified carcinoma (LC+OSC), and unspecified malignant neoplasms (unspecified). Race/ethnicity was classified as non-Hispanic White, non-Hispanic Black, Hispanic, and non-Hispanic Asian American/Pacific Islander; and are hereafter referred to as White, Black, Hispanic, and AAPI; respectively. Cases with unknown race/ethnicity were excluded (n = 587).

### Neighborhood factors

A previously developed composite score of neighborhood SES (nSES) was used based on principal component analysis of 2007–2011 American Community Survey (ACS) data on education, housing, employment, occupation, income, and poverty [15, 16]. Hispanic and Asian enclave indices were based on principal components analysis of 2007–2011 ACS variables related to Hispanic and Asian ethnic and immigrant composition and linguistic isolation, respectively [15, 16]. For both nSES and ethnic enclave, each census tract was assigned the appropriate composite score and then categorized in quartiles based on statewide distributions of scores for each factor. Lung cancer cases were assigned quartiles associated with the census tract of the geocoded residential address at diagnosis. A joint nSES+ ethnic enclave variable was created by combining each individual variable into two categories (i.e.; high nSES [quartiles 1–2], low nSES [quartiles 3–4], high enclave [quartile 4], and low enclave [quartiles 1–3), and categorizing the joint variable into: low nSES/high enclave; low nSES/low enclave; high nSES/high enclave; high nSES/low enclave.

### Population data

The calculation of 5-year incidence rates was based on the availability of the appropriate population denominators at the census tract level from 2010 Census data. The 2010 population counts were multiplied by five to estimate the total population at risk for the 5-year pericensal period of incidence, 2008–2012.

### Statistical analysis

Incidence rate ratios (IRRs) of lung cancer and 95% confidence intervals (CI) according to quartiles of nSES and Hispanic or Asian ethnic enclave status were estimated based on incidence rates per 100,000 and age-adjusted to the 2010 US Standard population. Stratified analyses were conducted to examine patterns of associations by sex and race/ethnicity. All analyses were conducted using SEER Stat, version 6.3.4 [17].

## Results

The distribution of neighborhood factors and clinical characteristics among the 75,631 lung cancer cases diagnosed from 2008–2012 are presented in Table 1. Approximately, 67% of Hispanics and 71% of Blacks resided in lower SES neighborhoods (first and second quartiles) in comparison to 41.9% of AAPIs and 42.3% of Whites. Proportionally more Hispanics and AAPIs resided in the highest enclave neighborhoods. Across all racial/ethnic groups combined, adenocarcinoma (47.5%) was the most common histologic cell-type.

**Table 1:** Distribution of neighborhood factors and clinical characteristics according to race/ethnicity among lung cancer cases, California 2008–2012.

			Race/ethnicity							
	Total		Hispanic		AAPI		Black		White	
	Male	Female	Male	Female	Male	Female	Male	Female	Male	Female
	N = 39,457	N = 36,174	N = 4406	N = 3889	N = 4915	N = 3634	N = 3258	N = 2704	N = 26,878	N = 25,947
	n	n	n	n	n	n	n	n	n	n
	(%)	(%)	(%)	(%)	(%)	(%)	(%)	(%)	(%)	(%)
**nSES**										
Q4-high	9546	9639	459	528	1373	1177	334	254	7380	7680
	(24.2%)	(26.6%)	(10.4%)	(13.6%)	(27.9%)	(32.4%)	(10.3%)	(9.4%)	(27.5%)	(29.6%)
Q3	10710	10056	863	878	1410	1008	665	526	7772	7644
	(27.1%)	(27.8%)	(19.6%)	(22.6%)	(28.7%)	(27.7%)	(20.4%)	(19.5%)	(28.9%)	(29.5%)
Q2	10843	9488	1283	1086	1290	878	910	807	7360	6717
	(27.5%)	(26.2%)	(29.1%)	(27.9%)	(26.2%)	(24.2%)	(27.9%)	(29.8%)	(27.4%)	(25.9%)
Q1-low	8358	6991	1801	1397	842	571	1349	1117	4366	3906
	(21.2%)	(19.3%)	(40.9%)	(35.9%)	(17.1%)	(15.7%)	(41.4%)	(41.3%)	(16.2%)	(15.1%)
**Ethnic enclave**^1^										
Q1-low	~	~	452	503	202	197	~	~	~	~
			(10.3%)	(12.9%)	(4.1%)	(5.4%)				
Q2	~	~	744	805	511	454	~	~	~	~
			(16.9%)	(20.7%)	(10.4%)	(12.5%)				
Q3	~	~	1257	1141	965	737	~	~	~	~
			(28.5%)	(29.3%)	(19.6%)	(20.3%)				
Q4- high	~	~	1953	1440	3237	2246	~	~	~	~
			(44.3%)	(37.0%)	(65.9%)	(61.8%)				
**Histologic cell-type**										
Adenocarcinoma	17126	18803	1897	2084	2584	2610	1386	1317	11259	12792
	(43.4%)	(52.0%)	(43.1%)	(53.6%)	(52.6%)	(71.8%)	(42.5%)	(48.7%)	(41.9%)	(49.3%)
SCC	9197	5617	982	544	920	290	769	475	6526	4308
	(23.3%)	(15.5%)	(22.3%)	(14.0%)	(18.7%)	(8.0%)	(23.6%)	(17.6%)	(24.3%)	(16.6%)
SCLC	4243	4102	472	406	412	148	282	296	3077	3252
	(10.8%)	(11.3%)	(10.7%)	(10.4%)	(8.4%)	(4.1%)	(8.7%)	(10.9%)	(11.4%)	(12.5%)
LC+OSC	2781	2798	374	364	268	175	219	209	1920	2050
	(7.0%)	(7.7%)	(8.5%)	(9.4%)	(5.5%)	(4.8%)	(6.7%)	(7.7%)	(7.1%)	(7.9%)
Unspecified	6109	4854	681	491	730	411	602	407	4096	3545
	(15.5%)	(13.4%)	(15.5%)	(12.6%)	(14.9%)	(11.3%)	(18.5%)	(15.1%)	(15.2%)	(13.7%)
**Age (years)**										
< 50	1371	1475	274	308	244	264	131	198	722	705
	(3.5%)	(4.1%)	(6.2%)	(7.9%)	(5.0%)	(7.3%)	(4.0%)	(7.3%)	(2.7%)	(2.7%)
50–59	5350	4753	610	602	679	601	717	557	3344	2993
	(13.6%)	(13.1%)	(13.8%)	(15.5%)	(13.8%)	(16.5%)	(22.0%)	(20.6%)	(12.4%)	(11.5%)
60–69	11456	9968	1181	1050	1348	920	1055	842	7872	7156
	(29.0%)	(27.6%)	(26.8%)	(27.0%)	(27.4%)	(25.3%)	(32.4%)	(31.1%)	(29.3%)	(27.6%)
70–79	13092	11967	1449	1206	1593	1136	968	761	9082	8864
	(33.2%)	(33.1%)	(32.9%)	(31.0%)	(32.4%)	(31.3%)	(29.7%)	(28.1%)	(33.8%)	(34.2%)
80+	8188	8011	892	723	1051	713	387	346	5858	6229
	(20.8%)	(22.1%)	(20.2%)	(18.6%)	(21.4%)	(19.6%)	(11.9%)	(12.8%)	(21.8%)	(24.0%)
**Stage**										
Localized	6602	7634	552	795	706	672	461	455	4883	5712
	(16.7%)	(21.1%)	(12.5%)	(20.4%)	(14.4%)	(18.5%)	(14.1%)	(16.8%)	(18.2%)	(22.0%)
Regional	8594	7870	886	789	993	701	709	607	6006	5773
	(21.8%)	(21.8%)	(20.1%)	(20.3%)	(20.2%)	(19.3%)	(21.8%)	(22.4%)	(22.3%)	(22.2%)
Distant	23121	19540	2815	2169	3043	2143	1995	1566	15268	13662
	(58.6%)	(54.0%)	(63.9%)	(55.8%)	(61.9%)	(59.0%)	(61.2%)	(57.9%)	(56.8%)	(52.7%)
Unstaged	1096	1068	149	127	166	111	92	74	689	756
	(2.8%)	(3.0%)	(3.4%)	(3.3%)	(3.4%)	(3.1%)	(2.8%)	(2.7%)	(2.6%)	(2.9%)

1. The ethnic enclave variable is defined only for Hispanic and Asian race/ethnicities.

2. AAPI, Asian American/Pacific Islander; nSES, neighborhood socioeconomic status; IRR, incidence rate ratio; CI, confidence interval; SCC, squamous cell carcinoma; SCLC, small cell lung cancer; LC+OSC, large cell and other cell lung cancer

Table 2 and S1 Fig present IRRs by nSES for overall lung cancer and histologic cell-types among males, stratified by racial/ethnic group. For overall lung cancer, Black, AAPI, and White male residents of lower SES neighborhoods had higher incidence of lung cancer compared to those in high SES neighborhoods. For adenocarcinoma, White male residents of lower SES neighborhoods had higher incidence (Q1 vs Q4: IRR, 1.42 [95% CI, 1.34–1.51]), and a similar pattern was suggested, although non-significant, among Black and AAPI males. In contrast, Hispanic male residents of lower SES neighborhoods, compared to high SES neighborhoods, had lower incidence of adenocarcinoma (Q1 vs. Q4: IRR, 0.86; [95% CI, 0.74–1.01]). For all other histologic cell-types (SCLC, SCC, LC+OSC) and unspecified lung cancers, Black, AAPI, and White male residents of lower SES neighborhoods had higher incidence (although IRRs were not significant for Black males with SCLC). For example, a greater than 2.5-fold incidence rate of SCC was observed in White males living in the lowest SES neighborhoods compared to the highest SES neighborhoods (Q1 vs. Q4: IRR, 2.81 [95% CI, 2.60–3.03]).

**Table 2:** Lung cancer incidence rate ratios^1^ for all lung cancers and histologic cell-types by nSES for males according to race/ethnicity, California 2008–2012.

		Hispanic			AAPI^2^			Black			White		
	nSES^2^	Cases	IRR^2^	(95% CI^2^)	Cases	IRR	(95% CI)	Cases	IRR	(95% CI)	Cases	IRR	(95% CI)
Overall lung cancer	Q4-high	459	1.00	reference	1373	1.00	reference	334	1.00	reference	7380	1.00	reference
	Q3	863	1.01	(0.90, 1.14)	1410	**1.19**	**(1.10, 1.28)**	665	**1.16**	**(1.00, 1.34)**	7772	**1.30**	**(1.26, 1.34)**
	Q2	1283	1.00	(0.89, 1.12)	1290	**1.26**	**(1.16, 1.36)**	910	**1.27**	**(1.11, 1.46)**	7360	**1.56**	**(1.51, 1.62)**
	Q1-low	1801	1.02	(0.92, 1.14)	842	**1.32**	**(1.20, 1.44)**	1349	**1.50**	**(1.32, 1.71)**	4366	**2.00**	**(1.92, 2.08)**
Adenocarcinoma	Q4-high	224	1.00	reference	803	1.00	reference	165	1.00	reference	3584	1.00	reference
	Q3	367	0.88	(0.74, 1.05)	747	1.08	(0.98, 1.20)	284	0.95	(0.77, 1.17)	3288	**1.13**	**(1.08, 1.19)**
	Q2	556	0.87	(0.74, 1.02)	643	1.09	(0.98, 1.21)	393	1.03	(0.85, 1.26)	2869	**1.25**	**(1.19, 1.31)**
	Q1-low	750	0.86	(0.74, 1.01)	391	1.07	(0.94, 1.21)	544	1.18	(0.98, 1.43)	1518	**1.42**	**(1.34, 1.51)**
SCC^2^	Q4-high	95	1.00	reference	223	1.00	reference	67	1.00	reference	1508	1.00	reference
	Q3	207	1.20	(0.93, 1.57)	252	**1.27**	**(1.05, 1.53)**	160	**1.50**	**(1.11, 2.07)**	1854	**1.53**	**(1.43, 1.64)**
	Q2	289	1.16	(0.91, 1.50)	260	**1.53**	**(1.27, 1.85)**	215	**1.64**	**(1.22, 2.24)**	1918	**2.01**	**(1.87, 2.15)**
	Q1-low	391	1.12	(0.88, 1.43)	185	**1.69**	**(1.38, 2.07)**	327	**1.93**	**(1.46, 2.60)**	1246	**2.81**	**(2.60, 3.03)**
SCLC^2^	Q4-high	41	1.00	reference	88	1.00	reference	31	1.00	reference	706	1.00	reference
	Q3	88	1.12	(0.76, 1.70)	123	**1.64**	**(1.23, 2.20)**	57	1.03	(0.63, 1.72)	912	**1.58**	**(1.43, 1.75)**
	Q2	130	1.12	(0.77, 1.66)	129	**1.97**	**(1.48, 2.64)**	78	1.19	(0.76, 1.95)	902	**2.03**	**(1.83, 2.24)**
	Q1-low	213	1.35	(0.95, 1.97)	72	**1.74**	**(1.25, 2.42)**	116	1.34	(0.87, 2.14)	557	**2.69**	**(2.40, 3.02)**
LC+OSC^2^	Q4-high	34	1.00	reference	69	1.00	reference	20	1.00	reference	558	1.00	reference
	Q3	75	1.19	(0.78, 1.88)	73	1.31	(0.92, 1.87)	47	1.43	(0.80, 2.64)	546	**1.20**	**(1.07, 1.36)**
	Q2	110	1.07	(0.71, 1.65)	77	**1.57**	**(1.11, 2.23)**	64	1.43	(0.83, 2.59)	506	**1.44**	**(1.27, 1.63)**
	Q1-low	155	1.11	(0.76, 1.70)	49	**1.66**	**(1.12, 2.46)**	88	**1.77**	**(1.05, 3.16)**	310	**1.89**	**(1.63, 2.18)**
Unspecified	Q4-high	65	1.00	reference	189	1.00	reference	51	1.00	reference	1024	1.00	reference
	Q3	126	1.01	(0.73, 1.40)	215	**1.26**	**(1.02, 1.55)**	117	1.38	(0.96, 2.03)	1172	**1.42**	**(1.30, 1.55)**
	Q2	198	1.11	(0.83, 1.52)	181	1.20	(0.97, 1.48)	160	**1.59**	**(1.12, 2.30)**	1165	**1.78**	**(1.63, 1.94)**
	Q1-low	292	1.16	(0.88, 1.57)	145	**1.55**	**(1.23, 1.94)**	274	**2.03**	**(1.46, 2.89)**	735	**2.42**	**(2.19, 2.67)**

1. Bold type indicates statistical significance

2. AAPI, Asian American/Pacific Islander; nSES, neighborhood socioeconomic status; IRR, incidence rate ratio; CI, confidence interval; SCC, squamous cell carcinoma; SCLC, small cell lung cancer; LC+OSC, large cell and other cell lung cancer

Among females (Table 3 and S2 Fig), Blacks and Whites residing in lower SES neighborhoods had higher incidence of overall lung cancer (Blacks Q1 vs. Q4: IRR, 1.43 [95% CI, 1.25–1.66] and Whites Q1 vs. Q4: IRR, 1.68 [95% CI, 1.61–1.75]) (Table 3). Conversely, Hispanic females residing in lower SES neighborhoods had lower incidence of overall lung cancer (Q1 vs. Q4: IRR, 0.79 [95% CI, 0.71–0.88]). For adenocarcinoma and LC+OSC, White females residing in lower SES neighborhoods, compared to high SES neighborhoods, had higher incidence, while Hispanic females residing in lower SES neighborhoods had lower incidence. For SCC and SCLC, Black, AAPI, and White females residing in lower SES neighborhoods, compared to high SES neighborhoods, had higher incidence.

**Table 3:** Lung cancer incidence rate ratios^1^ for all lung cancers and histologic cell-types by nSES for females according to race/ethnicity, California 2008–2012.

		Hispanic			AAPI^2^			Black			White		
	nSES^2^	Cases	IRR^2^	(95% CI^2^)	Cases	IRR	(95% CI)	Cases	IRR	(95% CI)	Cases	IRR	(95% CI)
Overall lung cancer	Q4-high	528	1.00	reference	1,177	1.00	reference	254	1.00	reference	7,680	1.00	reference
	Q3	878	0.95	(0.85, 1.06)	1,008	0.97	(0.89, 1.06)	526	1.09	(0.93, 1.27)	7,644	**1.18**	**(1.14, 1.22)**
	Q2	1,086	**0.81**	**(0.73, 0.91)**	878	1.00	(0.91, 1.09)	807	**1.31**	**(1.13, 1.52)**	6,717	**1.32**	**(1.27, 1.36)**
	Q1-low	1,397	**0.79**	**(0.71, 0.88)**	571	1.09	(0.98, 1.21)	1,117	**1.43**	**(1.25, 1.66)**	3,906	**1.68**	**(1.61, 1.75)**
Adenocarcinoma	Q4-high	320	1.00	reference	885	1.00	reference	148	1.00	reference	4,312	1.00	Reference
	Q3	487	0.87	(0.75, 1.00)	713	0.92	(0.83, 1.01)	268	0.94	(0.76, 1.17)	3,771	1.04	(0.99, 1.08)
	Q2	558	**0.69**	**(0.59, 0.79)**	624	0.94	(0.85, 1.05)	410	1.15	(0.94, 1.40)	3,116	**1.09**	**(1.04, 1.14)**
	Q1-low	719	**0.67**	**(0.59, 0.77)**	388	1.00	(0.88, 1.13)	491	1.08	(0.89, 1.31)	1,593	**1.23**	**(1.15, 1.30)**
SCC^2^	Q4-high	57	1.00	reference	72	1.00	reference	26	1.00	reference	1,010	1.00	Reference
	Q3	105	1.06	(0.76, 1.49)	79	1.21	(0.87, 1.71)	85	**1.80**	**(1.13, 2.96)**	1,285	**1.49**	**(1.37, 1.62)**
	Q2	166	1.12	(0.83, 1.56)	93	**1.72**	**(1.25, 2.39)**	135	**2.32**	**(1.50, 3.73)**	1,204	**1.77**	**(1.62, 1.93)**
	Q1-low	216	1.11	(0.82, 1.52)	46	1.35	(0.91, 2.00)	229	**3.06**	**(2.00, 4.84)**	809	**2.58**	**(2.34, 2.84)**
SCLC^2^	Q4-high	46	1.00	reference	37	1.00	reference	21	1.00	reference	730	1.00	Reference
	Q3	83	1.01	(0.70, 1.49)	40	1.12	(0.69, 1.82)	48	1.20	(0.70, 2.14)	938	**1.52**	**(1.38, 1.69)**
	Q2	115	1.01	(0.71, 1.47)	40	1.32	(0.82, 2.15)	86	1.60	(0.97, 2.75)	959	**1.96**	**(1.77, 2.17)**
	Q1-low	162	1.06	(0.75, 1.51)	31	**1.75**	**(1.04, 2.93)**	141	**2.07**	**(1.29, 3.50)**	625	**2.82**	**(2.53, 3.16)**
LC+OSC^2^	Q4-high	52	1.00	reference	55	1.00	reference	18	1.00	reference	623	1.00	Reference
	Q3	88	0.97	(0.67, 1.40)	50	1.06	(0.71, 1.60)	41	1.11	(0.62, 2.10)	595	**1.15**	**(1.02, 1.29)**
	Q2	108	0.82	(0.58, 1.17)	41	1.03	(0.67, 1.59)	59	1.27	(0.73, 2.32)	516	**1.26**	**(1.12, 1.43)**
	Q1-low	116	**0.68**	**(0.48, 0.96)**	29	1.21	(0.74, 1.97)	91	1.62	(0.96, 2.91)	316	**1.70**	**(1.47, 1.96)**
Unspecified	Q4-high	53	1.00	reference	128	1.00	reference	41	1.00	reference	1,005	1.00	Reference
	Q3	115	1.21	(0.86, 1.72)	126	1.09	(0.84, 1.41)	84	1.07	(0.72, 1.62)	1,055	**1.24**	**(1.13, 1.36)**
	Q2	139	1.02	(0.74, 1.44)	80	0.81	(0.60, 1.09)	117	1.13	(0.78, 1.68)	922	**1.39**	**(1.26, 1.52)**
	Q1-low	184	1.01	(0.74, 1.41)	77	1.28	(0.95, 1.72)	165	1.27	(0.89, 1.86)	563	**1.85**	**(1.67, 2.06)**

1. Bold type indicates statistical significance

2. AAPI, Asian American/Pacific Islander; nSES, neighborhood socioeconomic status; IRR, incidence rate ratio; CI, confidence interval; SCC, squamous cell carcinoma; SCLC, small cell lung cancer; LC+OSC, large cell and other cell lung cancer

Tables 4 and 5 and S3 and S4 Figs present IRRs of overall lung cancer and histologic cell-types by Hispanic and Asian ethnic enclave among Hispanics and AAPI, respectively. Among Hispanics residing in lower Hispanic enclave (more acculturated) neighborhoods compared to higher enclave neighborhoods (Table 4 and S3 Fig), higher incidence was observed for overall lung cancer and adenocarcinoma among both males and females (adenocarcinoma, Q1 vs. Q4: IRR, 1.24 [95% CI, 1.05–1.46] and IRR, 1.64 [95% CI, 1.42–1.89], respectively). For SCC and LC+OSC, similar patterns of higher incidence rates for those in lower enclave neighborhoods were observed among Hispanic females. Hispanic females residing in lower enclave neighborhoods, compared to high enclave neighborhoods, also had higher incidence of SCLC (Q1 vs. Q4: IRR, 1.68 [95% CI, 1.19–2.32]), but results suggested the opposite pattern of SCLC incidence among Hispanic males residing in lower enclave neighborhoods (Q3 vs. Q4: IRR, 0.72 [95% CI, 0.56–0.91]).

**Table 4:** Lung cancer incidence rate ratios^1^ for all lung cancers and histologic cell-types by Hispanic ethnic enclave for Hispanic males and females, California 2008–2012.

		Male			Female		
	Ethnic enclave	Cases	IRR^2^	(95% CI^2^)	Cases	IRR	(95% CI)
Overall lung cancer	Q4-high	1953	1.00	reference	1440	1.00	reference
	Q3	1257	1.02	(0.95, 1.10)	1141	**1.25**	**(1.15, 1.35)**
	Q2	744	1.07	(0.97, 1.17)	805	**1.50**	**(1.37, 1.63)**
	Q1-low	452	**1.14**	**(1.02, 1.27)**	503	**1.64**	**(1.48, 1.82)**
Adenocarcinoma	Q4-high	801	1.00	reference	767	1.00	reference
	Q3	569	**1.14**	**(1.01, 1.27)**	600	**1.23**	**(1.10, 1.37)**
	Q2	330	**1.18**	**(1.02, 1.35)**	446	**1.56**	**(1.38, 1.76)**
	Q1-low	197	**1.24**	**(1.05, 1.46)**	271	**1.64**	**(1.42, 1.89)**
SCC^2^	Q4-high	430	1.00	reference	197	1.00	reference
	Q3	276	1.02	(0.87, 1.19)	174	**1.42**	**(1.15, 1.76)**
	Q2	165	1.03	(0.85, 1.24)	110	**1.49**	**(1.17, 1.90)**
	Q1-low	111	1.20	(0.96, 1.49)	63	**1.58**	**(1.17, 2.11)**
SCLC^2^	Q4-high	237	1.00	reference	149	1.00	reference
	Q3	114	**0.72**	**(0.56, 0.91)**	116	1.17	(0.90, 1.50)
	Q2	83	0.96	(0.73, 1.25)	89	**1.56**	**(1.18, 2.05)**
	Q1-low	38	0.73	(0.49, 1.04)	52	**1.68**	**(1.19, 2.32)**
LC+OSC^2^	Q4-high	165	1.00	reference	128	1.00	reference
	Q3	117	1.26	(0.97, 1.63)	117	**1.48**	**(1.13, 1.93)**
	Q2	51	0.89	(0.62, 1.24)	68	**1.43**	**(1.04, 1.95)**
	Q1-low	41	1.42	(0.97, 2.03)	51	**1.93**	**(1.35, 2.70)**
Unspecified	Q4-high	320	1.00	reference	199	1.00	reference
	Q3	181	0.88	(0.72, 1.07)	134	1.06	(0.84, 1.34)
	Q2	115	1.01	(0.8, 1.27)	92	1.23	(0.95, 1.59)
	Q1-low	65	0.96	(0.71, 1.27)	66	**1.53**	**(1.13, 2.04)**

1. Bold type indicates statistical significance

2. IRR, incidence rate ratio; CI, confidence interval; SCC, squamous cell carcinoma; SCLC, small cell lung cancer; LC+OSC, large cell and other cell lung cancer

**Table 5:** Lung cancer incidence rate ratios^1^ for all lung cancers and histologic cell-types^2^ by Asian ethnic enclave for AAPI^3^ males and females, California 2008–2012.

		Male			Female		
	Ethnic enclave	Cases	IRR^3^	(95% CI^3^)	Cases	IRR	(95% CI)
Overall lung cancer	Q4-high	3237	1.00	reference	2246	1.00	reference
	Q3	965	0.96	(0.89, 1.03)	737	0.99	(0.91, 1.08)
	Q2	511	0.93	(0.84, 1.02)	454	1.08	(0.97, 1.20)
	Q1-low	202	0.93	(0.80, 1.08)	197	1.15	(0.99, 1.33)
Adenocarcinoma	Q4-high	1714	1.00	reference	1667	1.00	reference
	Q3	505	0.93	(0.84, 1.04)	520	0.93	(0.84, 1.02)
	Q2	263	0.91	(0.79, 1.04)	298	0.96	(0.84, 1.09)
	Q1-low	102	0.89	(0.72, 1.09)	125	0.98	(0.81, 1.18)
SCC^3^	Q4-high	591	1.00	reference	145	1.00	reference
	Q3	185	1.03	(0.87, 1.22)	73	**1.59**	**(1.18, 2.13)**
	Q2	99	1.01	(0.80, 1.25)	44	**1.68**	**(1.16, 2.37)**
	Q1-low	45	1.13	(0.80, 1.54)	28	**2.64**	**(1.69, 4.01)**
SCLC^3^	Q4-high	278	1.00	reference	79	1.00	reference
	Q3	72	0.83	(0.62, 1.08)	31	1.21	(0.76, 1.86)
	Q2	42	0.86	(0.60, 1.20)	20	1.32	(0.76, 2.21)
	Q1-low	20	0.99	(0.58, 1.58)	18	**2.86**	**(1.59, 4.91)**
Unspecified	Q4-high	467	1.00	reference	257	1.00	reference
	Q3	154	1.04	(0.86, 1.26)	70	0.84	(0.64, 1.11)
	Q2	82	1.00	(0.77, 1.27)	68	**1.36**	**(1.02, 1.79)**
	Q1-low	27	0.92	(0.59, 1.36)	16	0.80	(0.45, 1.35)

1. Bold type indicates statistical significance

2. Results not provided for LC+OSC due to low numbers

3. AAPI, Asian American/Pacific Islander; IRR, incidence rate ratio; CI, confidence interval; SCC, squamous cell carcinoma; SCLC, small cell lung cancer; LC+OSC, large cell and other cell lung cancer

Among AAPI males (Table 5 and S4 Fig), there were no differences in incidence of overall lung cancer or specific histologic cell-types according to Asian ethnic enclave. There were also no differences in incidence of overall lung cancer or adenocarcinoma according to Asian ethnic enclave among AAPI females. However, AAPI females residing in lower relative to higher enclave neighborhoods had substantially higher incidence of SCC and SCLC (Q1 vs. Q4: IRR, 2.64 [95%CI, 1.69–4.01] and IRR, 2.86 [95% CI, 1.59–4.91], respectively).

We also examined incidence of overall lung cancer and adenocarcinoma (the most common histologic cell-type) jointly by nSES and ethnic enclave (Table 6 and S5 Fig). For overall lung cancer, Hispanic males residing in low SES, low enclave neighborhoods had higher incidence compared to those in low SES, high enclave neighborhoods. On the other hand, Hispanic females residing in low enclave neighborhoods regardless of SES had higher overall lung cancer incidence than those residing in low SES, high enclave neighborhoods. For adenocarcinoma, Hispanic males and females, residing in both low nSES and high nSES neighborhoods that were low enclave had higher incidence of adenocarcinoma compared to those residing in low nSES, high enclave neighborhoods; males (IRR, 1.17 [95% CI, 1.04–1.32] and IRR, 1.15 [95% CI, 1.02–1.29], respectively) and females (IRR, 1.29 [95% CI, 1.15–1.44] and IRR, 1.51 [95% CI, 1.36–1.67], respectively). For overall lung cancer, only AAPI males residing in high SES, low enclave neighborhoods had lower incidence than those residing in low SES, high enclave neighborhoods (IRR, 0.81; 95% CI, 0.76–0.87). However, AAPI males residing in both low SES and high SES, low enclave neighborhoods had lower adenocarcinoma incidence. There were no differences in incidence of overall lung cancer or adenocarcinoma by the joint nSES/ethnic enclave measure among AAPI females. We were unable to examine incidence of other histologic cell types jointly by nSES and enclave status due to low numbers of cases.

**Table 6:** Lung cancer incidence rate ratios^1^ for all lung cancers and lung adenocarcinomas by joint nSES, ethnic enclave for Hispanic and AAPI males and females, California 2008–2012.

**Hispanic**							
		**Male**			**Female**		
	**nSES, ethnic enclave**^2^	**Cases**	**IRR**^3^	**(95% CI**^3^**)**	**Cases**	**IRR**	**(95% CI)**
Overall lung cancer	Low nSES, High enclave	1896	1.00		1390	1.00	
	Low nSES, Low enclave	1188	**1.09**	**(1.01, 1.18)**	1093	**1.41**	**(1.29, 1.53)**
	High nSES, High enclave	57	1.03	(0.77, 1.35)	50	1.14	(0.84, 1.51)
	High nSES, Low enclave	1265	1.03	(0.95, 1.11)	1356	**1.39**	**(1.29, 1.51)**
Adenocarcinoma	Low nSES, High enclave	784	1.00	reference	740	1.00	reference
	Low nSES, Low enclave	522	**1.17**	**(1.04, 1.32)**	537	**1.29**	**(1.15, 1.44)**
	High nSES, High enclave	17	0.79	(0.45, 1.27)	27	1.14	(0.74, 1.67)
	High nSES, Low enclave	574	**1.15**	**(1.02, 1.29)**	780	**1.51**	**(1.36, 1.67)**
**AAPI**^**3**^							
		**Male**			**Female**		
	**nSES, ethnic enclave**	**Cases**	**IRR**	**(95% CI)**	**Cases**	**IRR**	**(95% CI)**
Overall lung cancer	Low nSES, High enclave	1153	1.00		720	1.00	
	Low nSES, Low enclave	979	0.93	(0.86, 1.02)	729	1.05	(0.94, 1.16)
	High nSES, High enclave	122	1.10	(0.91, 1.34)	85	1.16	(0.91, 1.46)
	High nSES, Low enclave	2661	**0.81**	**(0.76, 0.87)**	2100	0.98	(0.89, 1.06)
Adenocarcinoma	Low nSES, High enclave	575	1.00		524	1.00	
	Low nSES, Low enclave	459	**0.88**	**(0.77, 0.99)**	488	0.96	(0.84, 1.09)
	High nSES, High enclave	61	1.10	(0.82, 1.44)	60	1.10	(0.83, 1.45)
	High nSES, Low enclave	1489	**0.90**	**(0.82, 0.99)**	1538	0.97	(0.88, 1.08)

1. Bold type indicates statistical significance.

2. Low nSES, quartiles 1–2; High SES, quartiles 3–4; High enclave, quartile 4; Low enclave, quartiles 1–3.

3. AAPI, Asian American/Pacific Islander; IRR, incidence rate ratio; CI, confidence interval.

## Discussion

In this population-based study with over 75,000 lung cancer cases, we observed higher incidence of lung cancer among Black and White males and females and AAPI males residing in lower SES neighborhoods. In contrast, incidence did not differ by nSES among AAPI females and Hispanic males, while an inverse association was observed among Hispanic females such that lung cancer incidence was actually lower in lower SES neighborhoods. However, examining histologic cell-type-specific lung cancer incidence according to nSES indicated somewhat varying patterns, especially among Hispanics and AAPIs. Among Hispanics and AAPIs, we also observed varying patterns of lung cancer incidence according to ethnic enclave that were specific to groups defined by sex, race/ethnicity, and histologic cell type. Examining incidence patterns jointly by nSES and ethnic enclave among Hispanics and AAPIs suggested that nSES-related gradients may, in some cases, be driven more by ethnic enclave status rather than by SES.

Previous studies have reported higher incidence of overall lung cancer among those of lower area-level SES [2–5] for most groups defined by sex and race/ethnicity, similar to our report. Higher incidence of overall lung cancer among Black and White males and females residing in lower SES neighborhoods likely reflects higher levels of smoking among Black and White residents of low SES neighborhoods [7, 18, 19]. Similar to previous studies, we also report lower incidence of overall lung cancer among Hispanic females residing in lower SES neighborhoods and null associations among AAPI females [2, 4, 5]. However, contrary to previous reports, we do not observe patterns of overall lung cancer incidence by nSES among Hispanic males [2, 4, 5]. This result from our contemporary population (2008–2012) may reflect changing associations over time as Wong et al., in a study of lung cancer cases from 1988–2002, reported a positive linear trend between nSES and NSCLC incidence among Hispanic males that was driven by older age groups [4].

The pattern of higher lung cancer incidence for residents of lower SES neighborhoods generally holds for most histologic cell-types among AAPI males and Black and White males and females. However, this pattern was least consistent for adenocarcinoma across races/ethnicities and the magnitude of effect was generally smaller. The association between smoking and adenocarcinoma is weaker compared to other histologic cell-types [12, 20], thus our results likely reflect other environmental exposures and genetic and molecular factors (that presumably are not as strongly associated with nSES as smoking) as the driving factors of adenocarcinoma development [12, 20]. In fact, with increasing understanding of the high rates of epidermal growth factor receptor (*EGFR*) mutated lung cancer in non-smoking Asian and Hispanic women, it is clear that the etiology of this subtype of lung cancer is quite different than the more common smoking-associated subtypes [21–23].

Previous studies have suggested that, compared to nSES, acculturation factors may be stronger predictors of lung cancer incidence among Hispanics [4, 11]. In a SEER study of lung cancers diagnosed among Hispanics (1988–1992), Eschbach et al. reported that Hispanic males and females residing in census tracts with less than 20% Hispanic populations had higher incidence of overall lung cancer [11]. Our results confirm this with a contemporary Hispanic population, using a multi-component Hispanic ethnic enclave measure [15, 24], and further show that the influence of Hispanic ethnic enclave on lung cancer incidence differs by sex and lung cancer histologic cell-type: lower Hispanic enclave was associated with greater incidence only for Hispanic males with adenocarcinoma, but across all histologic cell-types for Hispanic females. We found that low ethnic enclave drives patterns in lung adenocarcinoma incidence among Hispanic male and female residents, an effect that seemed particularly strong for Hispanic females. However, for overall lung cancer incidence, the observation that only Hispanic males residing in neighborhoods of both low nSES and low enclave had higher incidence of overall lung cancer indicates the importance of both nSES and ethnic enclave.

There is little information in the literature regarding associations between neighborhood acculturation and smoking prevalence among Hispanic adults. A study of Mexican adults reported null associations between neighborhood acculturation factors and smoking rates, but the multi-level model included a potential mediator, individual acculturation, which has been associated with increased rates of smoking among Hispanics, especially Hispanic females [25–27]. Furthermore, ethnic enclaves may influence smoking rates (for better or worse) via pathways both dependent and independent of individual acculturation. Additional potential mediators of enclave on smoking prevalence include the reinforcement of cultural norms [24, 28], discrimination and chronic stress [24], and targeted tobacco advertising [29]. In addition, the effects of ethnic enclaves on smoking rates among Hispanics likely differ by the dominant national origin within the neighborhood; [26, 30]. For example, Puerto Rican-Americans and Cuban-Americans were more likely to be heavy smokers than Mexican-Americans [30]. In California, the Hispanic population is predominantly of Mexican origin [31].

To our knowledge, no prior study has considered Asian ethnic enclave and lung cancer incidence among AAPIs. Examining incidence jointly by nSES and ethnic enclave among AAPI males indicates significantly lower incidence of adenocarcinoma for residents of low enclave neighborhoods, regardless of nSES, which suggests that high ethnic enclave drives adenocarcinoma incidence in this group. Among AAPI females, neighborhood SES and Asian enclave status may be less relevant to the etiology of adenocarcinoma and LC+OSC. Our power was limited to evaluate joint associations for other sub-types, where independent associations for nSES and enclave were much stronger, as was the case with SCC and SCLC histologic cell-types. To our knowledge, only one study by Kandula et al. addressed associations between neighborhood factors and smoking prevalence among Asians [32] and reported that, among Asian females, residing in a neighborhood with a lower percent of Asians was associated with a higher smoking prevalence. Therefore, our findings for the SCC and SCLC histologic cell-types, the two cell-types most strongly associated with smoking, may be due to higher smoking among AAPI females living in lower enclave neighborhoods [32].

Furthermore, any influence of ethnic enclave on smoking or other lung cancer risk factors among AAPIs is likely influenced by the dominant neighborhood culture, as ever-smoking prevalence among AAPI males differs greatly by national origin, ranging from 15% among Vietnamese males to 59% among Native Hawaiian/Pacific Islander males [33]. In addition, ever-smoking prevalence varies widely according to national origin among Asian females, ranging from 0.3% in Vietnamese to 40% in Japanese females [33]. Thus, future studies should consider smoking status in addition to nSES, ethnic enclave, and histologic sub-type among AAPIs, as well as associations between neighborhood factors and lung cancer incidence across specific AAPI subgroups.

There are several strengths to our study. Our study included a large population-based sample of diverse lung cancer cases with sufficient numbers to address subtypes separately and utilized composite indices for measuring nSES and neighborhood acculturation [15]. As a result, we were able to uncover undocumented associations between neighborhood factors and lung cancer incidence. However, our study does have some limitations. We were unable to disaggregate AAPIs and Hispanics into specific populations defined by national origin or immigration status, due to lack of appropriate population denominator data. In addition, we were unable to evaluate clinical factors (such as tumor associated mutations), individual-level factors such as smoking history, passive smoking, family history of lung cancer, use of medical services, environmental exposures, or individual measures of SES or acculturation due to lack of availability of this data. Estimates of neighborhood-level smoking prevalences are not available, but would provide a clearer picture of neighborhood factors that correlate with aggregate smoking behavior. Finally, individuals’ residential history would improve the study of neighborhood factors associated with lung cancer risk, as it would provide a more accurate portrait of the lung cancer risk period.

## Conclusion

Our study illustrates varying patterns of incidence of lung cancer histologic cell types according to neighborhood SES and ethnic enclave by sex and race/ethnicity. It is important for continuing research on lung cancer etiology to consider, in addition to individual-level exposures (e.g.; smoking status, individual-level SES), the clear contextual-level effects of nSES and, for Hispanics and AAPIs, the joint effects of nSES and ethnic enclave.

## Supporting information

S1 Fig Lung cancer incidence rate ratios (IRRs) and 95% confidence intervals (95% CIs) according to neighborhood SES (nSES) quartile for lung cancer histologic cell-types among males diagnosed in California 2008–2012. IRRs and 95% CIs for (A) overall lung cancer, (B) adenocarcinoma, (C) squamous cell carcinoma (SCC), (D) small-cell lung carcinoma (SCLC), (E) large-cell and other specified carcinoma (LC+OSC), and (F) unspecified lung cancers among non-Hispanic White (White, orange), non-Hispanic Black (Black, green), Asian American and Pacific Islander (AAPI, red), and Hispanic (blue) males. Markers represent IRRs and horizontal solid lines represent 95% CIs. The highest nSES quartile (Q4) serves as the reference category (IRR, 1.0; represented by the vertical dotted line).
(DOCX)

S2 Fig Lung cancer incidence rate ratios (IRRs) and 95% confidence intervals (95% CIs) according to neighborhood SES (nSES) quartile for lung cancer histologic cell-types among females diagnosed in California 2008–2012. IRRs and 95% CIs for (A) overall lung cancer, (B) adenocarcinoma, (C) squamous cell carcinoma (SCC), (D) small-cell lung carcinoma (SCLC), (E) large-cell and other specified carcinoma (LC+OSC), and (F) unspecified lung cancers among non-Hispanic White (White, orange), non-Hispanic Black (Black, green), Asian American and Pacific Islander (AAPI, red), and Hispanic (blue) females. Markers represent IRRs and horizontal solid lines represent 95% CIs. The highest nSES quartile (Q4) serves as the reference category (IRR, 1.0; represented by the vertical dotted line).
(DOCX)

S3 Fig Lung cancer incidence rate ratios (IRRs) and 95% confidence intervals (95% CIs) according to quartile of neighborhood Hispanic ethnic enclave for lung cancer histologic cell-types among Hispanic males and females diagnosed in California 2008–2012. IRRs and 95% CIs for overall lung cancer (black), adenocarcinoma (blue), squamous cell carcinoma (SCC, red), small-cell lung carcinoma (SCLC, green), large-cell and other specified cell carcinoma (LC+OSC, orange), and unspecified lung cancers (purple) among Hispanic (A) males and (B) females. Markers represent IRRs and horizontal solid lines represent 95% CIs. The highest quartile of neighborhood Hispanic ethnic enclave (Q4) serves as the reference category (IRR, 1.0, represented by the vertical dotted line).
(DOCX)

S4 Fig Lung cancer incidence rate ratios (IRRs) and 95% confidence intervals (95% CIs) according to quartile of neighborhood Asian ethnic enclave for lung cancer histologic cell types among Asian American and Pacific Islander (AAPI) males and females diagnosed in California 2008–2012. IRRs and 95% CIs for overall lung cancer (black), adenocarcinoma (blue), squamous cell carcinoma (SCC, red), small-cell lung carcinoma (SCLC, green), large-cell and other specified cell carcinoma (LC+OSC, orange), and unspecified lung cancers (purple) among AAPI (A) males and (B) females. Markers represent IRRs and horizontal solid lines represent 95% CIs. The highest quartile of neighborhood Asian ethnic enclave (Q4) serves as the reference category (IRR, 1.0, represented by the vertical dotted line).
(DOCX)

S5 Fig Lung cancer incidence rate ratios (IRRs) and 95% confidence intervals (95% CIs) according to joint neighborhood socioeconomic status (nSES) and ethnic enclave among Hispanic and Asian American Pacific Islander (AAPI) males and females diagnosed in California 2008–2012. IRRs and 95% CIs for nSES/Hispanic ethnic enclave for all lung cancer (blue) and adenocarcinoma (red) among (A) Hispanic males and (B) Hispanic females and according to joint nSES/Asian ethnic enclave among (C) AAPI males and (D) AAPI females. Markers represent IRRs and horizontal solid lines represent 95% CIs. Low nSES/High enclave serves as the reference category (IRR, 1.0).
(DOCX)
