# Supplementary material for: Incidence of lung cancer histologic cell-types according to neighborhood factors: A population based study in California
Source: PLoS One. 2018 May 23;13(5):e0197146. doi: 10.1371/journal.pone.0197146 (PMC5965814; doi:10.1371/journal.pone.0197146)
Supplement: S1 Fig — IRRs and 95% CIs for (A) overall lung cancer, (B) adenocarcinoma, (C) squamous cell carcinoma (SCC), (D) small-cell lung carcinoma (SCLC), (E) large-cell and other specified carcinoma (LC+OSC), and (F) unspecified lung cancers among non-Hispanic White (White, orange), non-Hispanic Black (Black, green), Asian American and Pacific Islander (AAPI, red), and Hispanic (blue) males. Markers represent IRRs and horizontal solid lines represent 95% CIs. The highest nSES quartile (Q4) serves as the reference category (IRR, 1.0; represented by the vertical dotted line). (DOCX) [file pone.0197146.s001.docx]

**S1 Fig. Lung cancer incidence rate ratios (IRRs) and 95% confidence intervals (95% CIs) according to neighborhood SES (nSES) quartile for lung cancer histologic cell-types among males diagnosed in California 2008-2012**.

Hispanic

AAPI

Black

White

**D.**

**E.**

**F.**

Incidence Rate Ratio (95% CI)

Incidence Rate Ratio (95% CI)

**A.**

**B.**

**C.**

Lowest nSES

Lower-middle nSES

Higher-middle nSES

Lowest nSES

Lower-middle nSES

Higher-middle nSES

Lowest nSES

Lower-middle nSES

Higher-middle nSES

Lowest nSES

Lower-middle nSES

Higher-middle nSES

White

Black

AAPI

Hispanic

Lowest nSES

Lower-middle nSES

Higher-middle nSES

Lowest nSES

Lower-middle nSES

Higher-middle nSES

Lowest nSES

Lower-middle nSES

Higher-middle nSES

Lowest nSES

Lower-middle nSES

Higher-middle nSES


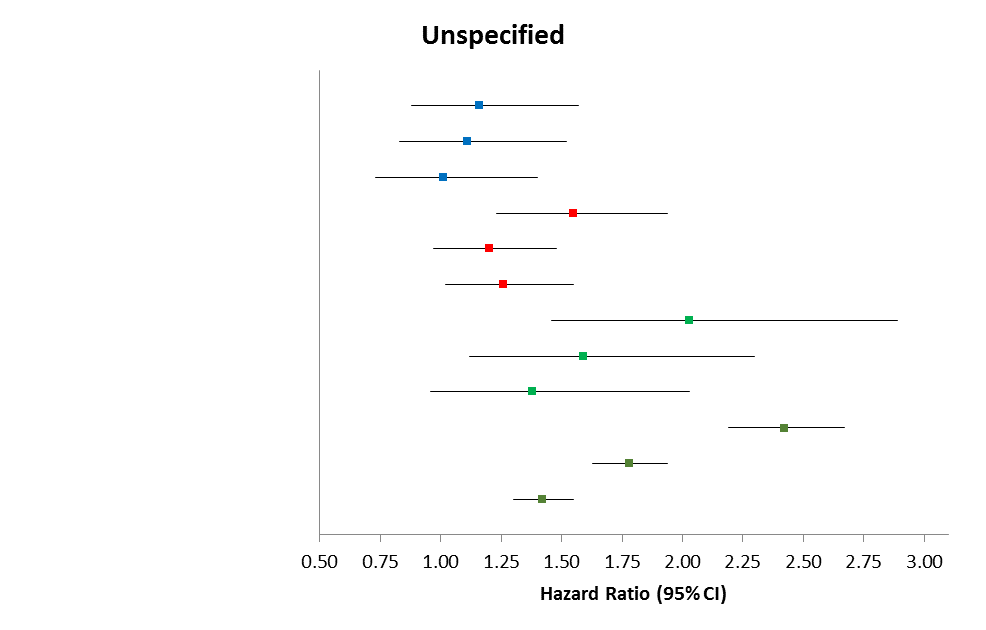


Lowest SES

Lower-middle SES

Higher-middle SES

Lowest SES

Lower-middle SES

Higher-middle SES

Lowest SES

Lower-middle SES

Higher-middle SES

Lowest SES

Lower-middle SES

Higher-middle SES

Hispanic

AAPI

Black

White


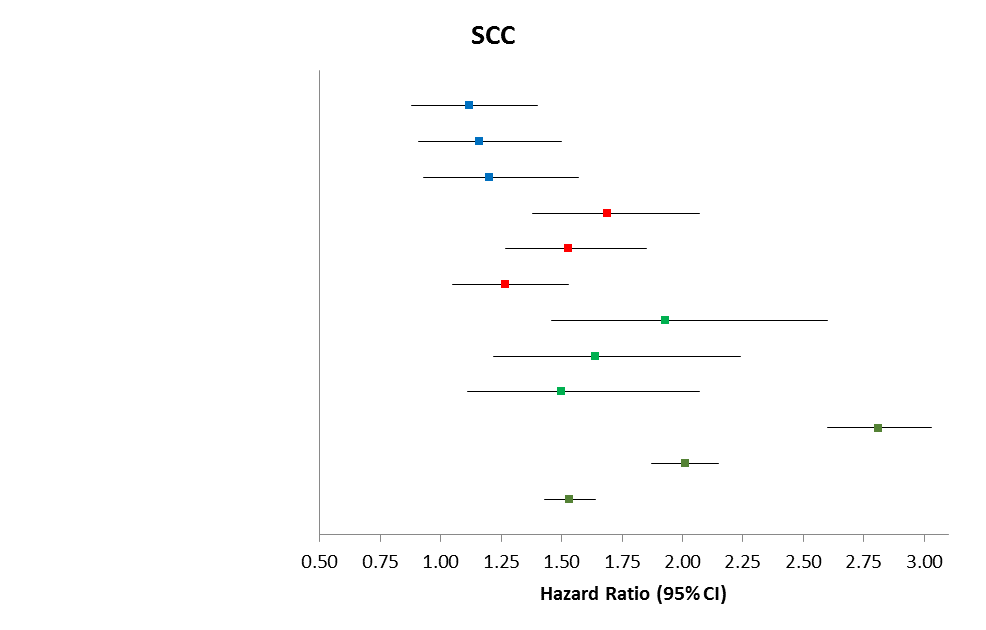


Lowest SES

Lower-middle SES

Higher-middle SES

Lowest SES

Lower-middle SES

Higher-middle SES

Lowest SES

Lower-middle SES

Higher-middle SES

Lowest SES

Lower-middle SES

Higher-middle SES

Hispanic

AAPI

Black

White
