# Supplementary material for: Incidence of lung cancer histologic cell-types according to neighborhood factors: A population based study in California
Source: PLoS One. 2018 May 23;13(5):e0197146. doi: 10.1371/journal.pone.0197146 (PMC5965814; doi:10.1371/journal.pone.0197146)
Supplement: S2 Fig — IRRs and 95% CIs for (A) overall lung cancer, (B) adenocarcinoma, (C) squamous cell carcinoma (SCC), (D) small-cell lung carcinoma (SCLC), (E) large-cell and other specified carcinoma (LC+OSC), and (F) unspecified lung cancers among non-Hispanic White (White, orange), non-Hispanic Black (Black, green), Asian American and Pacific Islander (AAPI, red), and Hispanic (blue) females. Markers represent IRRs and horizontal solid lines represent 95% CIs. The highest nSES quartile (Q4) serves as the reference category (IRR, 1.0; represented by the vertical dotted line). (DOCX) [file pone.0197146.s002.docx]

**S2 Fig. Lung cancer incidence rate ratios (IRRs) and 95% confidence intervals (95% CIs) according to neighborhood SES (nSES) quartile for lung cancer histologic cell-types among females diagnosed in California 2002-2012**

Incidence Rate Ratio (95% CI)

Incidence Rate Ratio (95% CI)

Lowest nSES

Lower-middle nSES

Higher-middle nSES

Lowest nSES

Lower-middle nSES

Higher-middle nSES

Lowest nSES

Lower-middle nSES

Higher-middle nSES

Lowest nSES

Lower-middle nSES

Higher-middle nSES

Lowest nSES

Lower-middle nSES

Higher-middle nSES

Lowest nSES

Lower-middle nSES

Higher-middle nSES

Lowest nSES

Lower-middle nSES

Higher-middle nSES

Lowest nSES

Lower-middle nSES

Higher-middle nSES

**C.**

**F.**

**E.**

**B.**

**D.**

**A.**

White

Black

AAPI

Hispanic

White

Black

AAPI

Hispanic


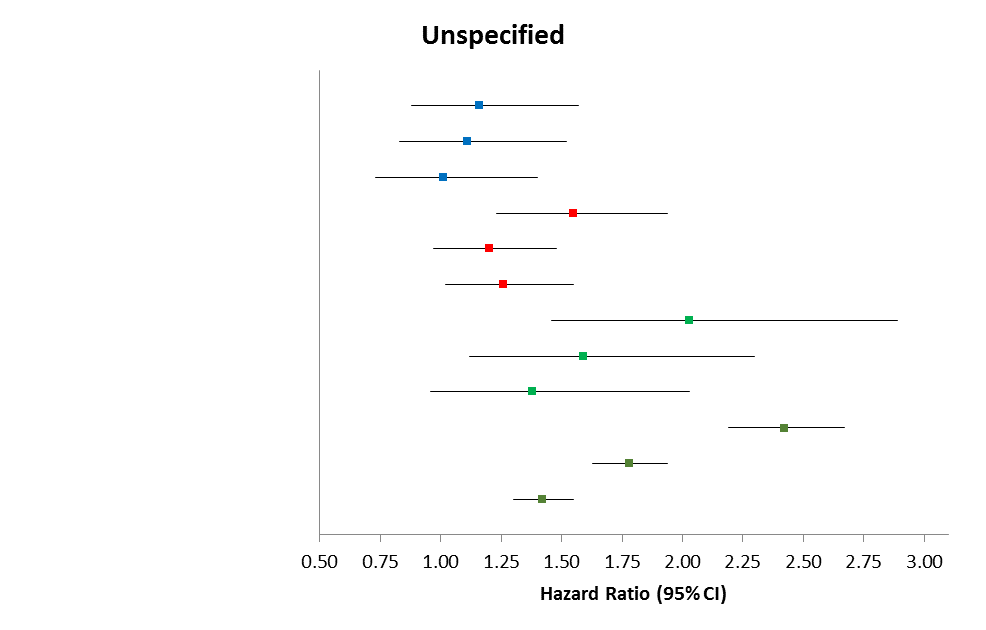


Lowest SES

Lower-middle SES

Higher-middle SES

Lowest SES

Lower-middle SES

Higher-middle SES

Lowest SES

Lower-middle SES

Higher-middle SES

Lowest SES

Lower-middle SES

Higher-middle SES

Hispanic

AAPI

Black

White


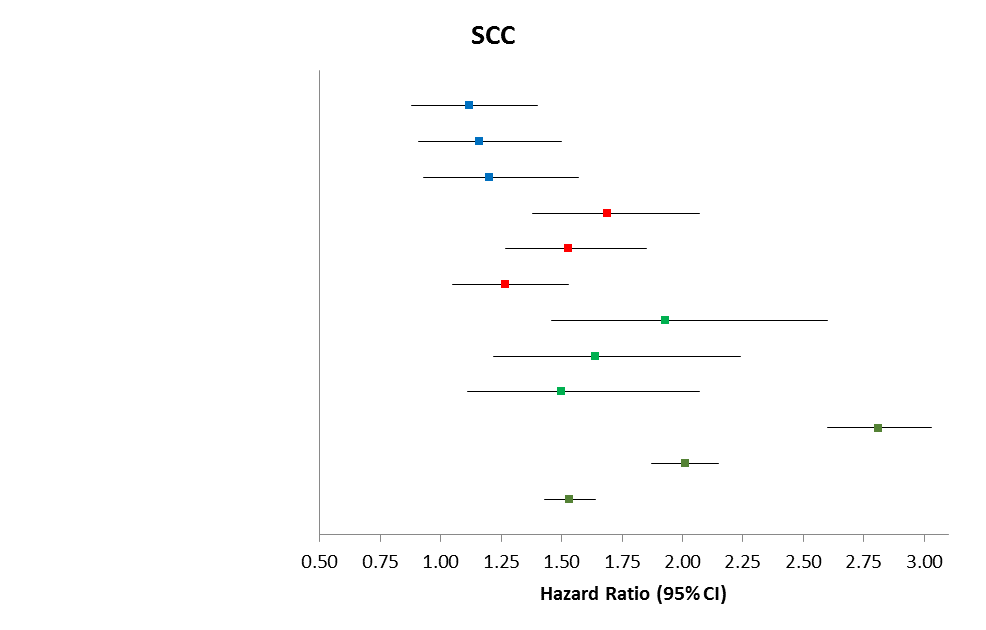


Lowest SES

Lower-middle SES

Higher-middle SES

Lowest SES

Lower-middle SES

Higher-middle SES

Lowest SES

Lower-middle SES

Higher-middle SES

Lowest SES

Lower-middle SES

Higher-middle SES

Hispanic

AAPI

Black

White
