# Supplementary material for: Incidence of lung cancer histologic cell-types according to neighborhood factors: A population based study in California
Source: PLoS One. 2018 May 23;13(5):e0197146. doi: 10.1371/journal.pone.0197146 (PMC5965814; doi:10.1371/journal.pone.0197146)
Supplement: S3 Fig — IRRs and 95% CIs for overall lung cancer (black), adenocarcinoma (blue), squamous cell carcinoma (SCC, red), small-cell lung carcinoma (SCLC, green), large-cell and other specified cell carcinoma (LC+OSC, orange), and unspecified lung cancers (purple) among Hispanic (A) males and (B) females. Markers represent IRRs and horizontal solid lines represent 95% CIs. The highest quartile of neighborhood Hispanic ethnic enclave (Q4) serves as the reference category (IRR, 1.0, represented by the vertical dotted line). (DOCX) [file pone.0197146.s003.docx]

**S3 Fig. Lung cancer incidence rate ratios (IRRs) and 95% confidence intervals (95% CIs) according to quartile of neighborhood Hispanic ethnic enclave for lung cancer histologic cell-types among Hispanic males and females diagnosed in California 2008-2012.**

Lowest enclave

Lower-middle enclave

Higher-middle enclave

Lowest enclave

Lower-middle enclave

Higher-middle enclave

Lowest enclave

Lower-middle enclave

Higher-middle enclave

Lowest enclave

Lower-middle enclave

Higher-middle enclave

Lowest enclave

Lower-middle enclave

Higher-middle enclave

Lowest enclave

Lower-middle enclave

Higher-middle enclave

Unspecified

LC+OSC

SCLC

SCC

Adenocarcinoma

Overall

lung cancer

**A.**

**B.**
