# Supplementary material for: Incidence of lung cancer histologic cell-types according to neighborhood factors: A population based study in California
Source: PLoS One. 2018 May 23;13(5):e0197146. doi: 10.1371/journal.pone.0197146 (PMC5965814; doi:10.1371/journal.pone.0197146)
Supplement: S5 Fig — IRRs and 95% CIs for nSES/Hispanic ethnic enclave for all lung cancer (blue) and adenocarcinoma (red) among (A) Hispanic males and (B) Hispanic females and according to joint nSES/Asian ethnic enclave among (C) AAPI males and (D) AAPI females. Markers represent IRRs and horizontal solid lines represent 95% CIs. Low nSES/High enclave serves as the reference category (IRR, 1.0). (DOCX) [file pone.0197146.s005.docx]

**S5 Fig. Lung cancer incidence rate ratios (IRRs) and 95% confidence intervals (95% CIs) according to joint neighborhood socioeconomic status (nSES) and ethnic enclave among Hispanic and Asian American Pacific Islander (AAPI) males and females diagnosed in California 2008-2012**.

**A.**

Overall

lung cancer

Adenocarcinoma

High nSES, Low enclave

High nSES, High enclave

Low nSES, Low enclave

Low nSES, High enclave

High nSES, Low enclave

High nSES, High enclave

Low nSES, Low enclave

Low nSES, High enclave

**B.**

High nSES, Low enclave

High nSES, High enclave

Low nSES, Low enclave

Low nSES, High enclave

High nSES, Low enclave

High nSES, High enclave

Low nSES, Low enclave

Low nSES, High enclave

Overall

lung cancer

Adenocarcinoma

Incidence rate ratio (95% CI)

**C.**

**D.**

Incidence rate ratio (95% CI)
